# Outcomes post kidney transplantation amongst First Nations Australians in the Northern Territory

**DOI:** 10.3389/fneph.2025.1677030

**Published:** 2025-10-09

**Authors:** Farhan Ali Khan, Katherine A. Barraclough, Sandawana William Majoni, Sajan Thomas, Wathsala Munasinghe, Asanga Abeyaratne, Robert Carroll

**Affiliations:** ^1^ Department of Nephrology, Royal Darwin Hospital, Darwin, NT, Australia; ^2^ Department of Nephrology, Royal Melbourne Hospital, Melbourne, VIC, Australia; ^3^ Department of Medicine, University of Melbourne, Melbourne, VIC, Australia; ^4^ Flinders University and Northern Territory Medical Program, Darwin, NT, Australia; ^5^ Wellbeing and Preventable Chronic Diseases, Menzies School of Health Research, Charles Darwin University, Darwin, NT, Australia; ^6^ Department of Nephrology, Alice Springs Hospital, Alice Springs, NT, Australia; ^7^ Lifeblood Adelaide, Adelaide, SA, Australia; ^8^ South Australian Transplant Immunogenetics Laboratory, LifeBlood, Women and Children’s Hospital, Adelaide, SA, Australia

**Keywords:** kidney, transplantation, indigenous, first nation, eplet matching, PIRCHE, outcomes, survival

## Abstract

**Aims:**

1)To compare graft and patient survival rates following deceased donor kidney transplantation in Northern Territory (NT) First Nations Australians between 2001–2011 and 2012-2021. 2)To compare transplant outcomes between First Nations and non-Indigenous Australians during 2012-2021. 3)To assess the impact of eplet mismatches and predicted indirectly recognizable HLA epitopes II (PIRCHE) scores on transplant outcomes in First Nations Australians.

**Background:**

Despite advancements in transplant outcomes across Australia, uncertainty exists regarding improvements in graft and patient survival rates for NT First Nations Australians. No study has evaluated the impact of molecular matching on post-transplant outcomes for NT First Nations Australians.

**Methods:**

We performed a retrospective cohort study involving NT First Nations Australians transplanted between 2001-2021. Participants were divided into two groups: 2001–2011 and 2012-2021. For comparison, we also included non-Indigenous recipients transplanted during the 2012–2021 period. We analyzed graft and patient survival using Kaplan-Meier curves and assessed the association of eplets and PIRCHE scores with graft outcomes and *de novo* donor specific antibody (dnDSA) formation.

**Results:**

Five-year graft and patient survival rates were 46% and 66% in the 2001–2011 cohort compared with 69.7% and 83.1% in the 2012–2021 cohort. For non-Indigenous recipients (2012-2021), 5-year graft and patient survival were 90.5% and 97.6%. Higher eplet mismatch loads and PIRCHE scores were not associated with graft survival, patient survival, or time to rejection among First Nations Australians.

**Conclusion:**

Post-transplant outcomes for First Nations Australians have improved considerably, but they remain inferior to non-Indigenous Australians.

## Introduction

Chronic kidney disease (CKD) is a leading cause of comorbidity and mortality worldwide, affecting greater than 10% of the population, which amounts to more than 800 million people (1). First Nations Australians are disproportionally affected by CKD, experiencing kidney failure and kidney replacement therapy (KRT) rates that are 6- and 8-fold higher than those of non-Indigenous Australians (2). The median age at which First Nations Australians reach kidney failure is up to 30 years lower than that of their non-Indigenous counterparts (2). First Nations Australians comprise approximately 3.8% of the total Australian population, with the Northern Territory (NT) having the highest proportion (31%) (3).

Historically, kidney transplant rates and outcomes for First Nations Australians have been poor (4–6). First Nations Australians often wait longer on dialysis, have more sensitizing events and increased infective complications. In turn, such factors contribute to lower graft and patient survival rates (7, 8). While kidney transplantation outcomes have improved across Australia over the last two decades, it is unclear whether these same advancements have been realized by First Nations Australians in the NT, which has the highest rate of prevalent First Nations transplant recipients amongst any state or territory (9).

First Nations Australians with a kidney transplant are also likely to have greater human leucocyte antigen (HLA) mismatches than non-Indigenous Australians, as the donor pool predominantly consists of individuals with European ancestry (10). Over the last decade, HLA typing has shifted from serological to molecular techniques, enabling a more comprehensive assessment of organ compatibility. High molecular mismatches have been associated with inferior post-transplant outcomes (11). High resolution HLA incompatibility scoring algorithms such as HLA-Matchmaker and predicted indirectly recognizable HLA epitopes II (PIRCHE) can predict *de novo* donor specific antibodies (dnDSA) development and graft failure in the general transplant population (12, 13). However, the impact of eplet mismatches and PIRCHE scores on transplant outcomes for First Nations Australian transplant recipients remains unclear.

Accordingly, the aims of this study were threefold: 1) to compare graft and patient survival rates following deceased donor (DD) kidney transplantation in First Nations Australians in the Northern Territory between 2001–2011 and 2012-2021; 2) to compare transplant outcomes between First Nations Australians and non-Indigenous Australians during the 2012–2021 period; and 3) to assess the impact of eplet mismatch loads and PIRCHE scores on the development of dnDSA, time to rejection, and graft survival in First Nations Australians between 2012-2021.

## Materials and methods

### Patient population

A retrospective analysis was conducted involving all First Nations Australians from the NT who received a deceased donor kidney transplant between 1^st^ January 2001 and 31st December 2021. Recipients were divided into two cohorts: those who received a transplant before 2012 and those who received one after. Non-Indigenous recipients who underwent transplantation between 1^st^ January 2012 and 31^st^ December 2021 were also included to enable a comparison of outcomes between First Nations and non-Indigenous Australians. The data censoring dates for the 2001–2011 and 2012–2021 cohorts were 31^st^ December 2012 and 31^st^ December 2022, respectively.

### Data collection

Individuals who received a DD kidney transplant between the specified dates were identified via the Australian OrganMatch database and verified against the NT Renal Service database. Baseline demographics and details of graft and patient outcomes were obtained from the NT Renal Service database and review of medical records. Eplet mismatch loads, PIRCHE scores and time to dnDSA formation were provided by the South Australian Transplantation and Immunogenetics Service. Donors and recipients did not under high resolution tissue typing under recently in Australia, and thus Eplet mismatch loads and PIRCHE scores were only available in the 2012–2021 cohort.

### Ethics

Ethics approval was obtained from the Northern Territory Health and Menzies School of Health Research Human Research Ethics Committees (approval number NT HREC, 2019-3249).

### Statistical analysis

Graft survival was calculated from the time of transplant to loss of graft. Graft loss due to patient death was included in the graft survival analysis but censored at the time of death in the death censored graft survival analysis, if graft loss didn’t result in patient death. Patient survival was calculated from time of transplant to death. Patients which were lost to follow up were excluded from further analysis.

After 2016, calculated panel reactive antibody (cPRA) values were derived from combined class I and II Luminex antibody profiles rather than cell dependent cytotoxicity (CDC) assays. This change increased the number of waitlisted people gaining preferential allocation based on HLA-A, -B and -DR matching and cPRA >50% and >80%. In early 2021, allocation criteria were further revised to prioritize waitlisted patients with cPRA >95%. Given the potential impact of these changes on First Nations Australians who carry distinct HLA haplotypes, we also compared the HLA and molecular mismatches in transplant recipients before and after 2016.

Survival analysis was conducted using standard time to event with Kaplan-Meier curves. The Z-test was used to determine if a significant difference in proportions was present in the populations. Multivariable conditional logistic regression for matched individuals was performed. The Mann-Whitney U test was conducted to compare PIRCHE scores and eplet mismatches. A Receiver Operator Characteristic (ROC) curve was conducted to determine PIRCHE scores and eplet mismatch thresholds best associated with dnDSA formation.

A two tailed p-value of less than 0.05 was considered significant.

## Results

Between January 2001 and December 2011, 50 First Nations Australians underwent deceased donor kidney transplantation in the NT. This number increased to 89 from January 2012 to December 2021. During the same period, 42 non-Indigenous Australians received a deceased donor kidney transplant.

Baseline characteristics of study participants are presented in **Table 1**. In the later era, First Nations Australians exhibited a higher rate of diabetic nephropathy as the primary kidney disease and a greater prevalence of pre-transplant diabetes (56.2% vs. 34%, p=0.012 and 69.7% vs. 48%, p=0.011, respectively) compared with the earlier era. Basiliximab was more commonly used for induction in the earlier era (96% vs. 47.2%, p<0.001), while ATG was more commonly used in the later era (4% vs. 52.8%, p<0.001). First Nations Australians in the later era showed worse HLA matching (p=0.004), with 69.7% of patients having a completely HLA mismatched graft.

**Table 1:** Baseline characteristics.

Characteristic	First nations Australians			Non-indigenous Australians	
	2001-2011 (n=50)	2012-2021 (n=89)	p*	2012-2021 (n=42)	p**
Male	32 (64%)	49 (55%)	.303	25 (59.5%)	.631
Age	48 (42-52.75)	48 (39-56)		53 (41.25-60.5)	
Primary kidney disease					
Diabetes	17 (34.0%)	50 (56.2%)	.012	5 (11.9%)	<.001
Glomerulonephritis	18 (36.0%)	10 (11.2%)	<.001	13 (30.1%)	.056
Unclear	11 (22.0%)	14 (15.7%)	.358	3 (7.1%)	.171
Other	4 (8.0%)	15 (16.9%)	.144	22 (52.4%)	<.001
Diabetes/IHD					
Diabetic pretransplant	24 (48.0%)	62 (69.7%)	.011	8 (19.0%)	<.001
IHD pretransplant	12 (24.0%)	20 (22.5%)	.834	6 (14.8%)	.271
NODAT	7 (14.0%)	8 (9.0%)	.362	7 (16.7%)	.197
Previous dialysis modality					
Hemodialysis	35 (70.0%)	66 (74.2%)	.596	19 (45.2%)	.001
Peritoneal dialysis	2 (4.0%)	4 (4.5%)	.889	4 (9.5%)	.263
Both	13 (26.0%)	19 (21.3%)	.529	19 (45.2%)	.005
Induction immunosuppression					
ATG	2 (4%)	47 (52.8%)	<.001	19 (45.2%)	.418
Basiliximab	48 (96%)	42 (47.2%)	<.001	23 (54.8%)	.418
HLA mismatches					
1	0 (0%)	0		3 (7.1%)	
2	1 (2.0%)	1 (1.1%)		6 (14.3%)	
3	1 (2.0%)	0		6 (14.3%)	
4	4 (8.0%)	11 (12.4%)		7 (16.7%)	
5	19 (38.0%)	15 (16.9%)		15 (35.7%)	
6	23 (46.0%)	62 (69.7%)	.006	5 (11.9%)	<.001
Donor age	–	50 (38.5-61)		48 (35-61)	
Eplet MM load	–	77 (25)		53 (27)	<.001
PIRCHE score	–	407 (172)		289 (174)	<.001

Results are presented as frequencies and percentages apart from median (interquartile range) for age and mean (standard deviation) for eplet MM load and PIRCHE score.

ATG, Anti-thymocyte Globulin; IHD, Ischemic Heart Disease; NODAT, New Onset Diabetes After Transplant; HLA, Human Leukocyte Antigen; MM, Mismatch; PIRCHE, Predicted Indirectly Recognizable HLA Epitopes.

*Comparison between First Nations transplant recipients in the 2 eras.

**Comparison between First Nations and non-Indigenous transplant recipients in the 2012–2021 era.

Among First Nations Australians, the mean number of hospitalizations in the first 2 years post-transplant increased across the 2 eras. However, the length of stay was lower. The rate of rejection in the first 2 years post-transplant fell over time (50% in 2001–2011 vs. 32.6% in 2012-2021, p=0.043).

**Figure 1:**
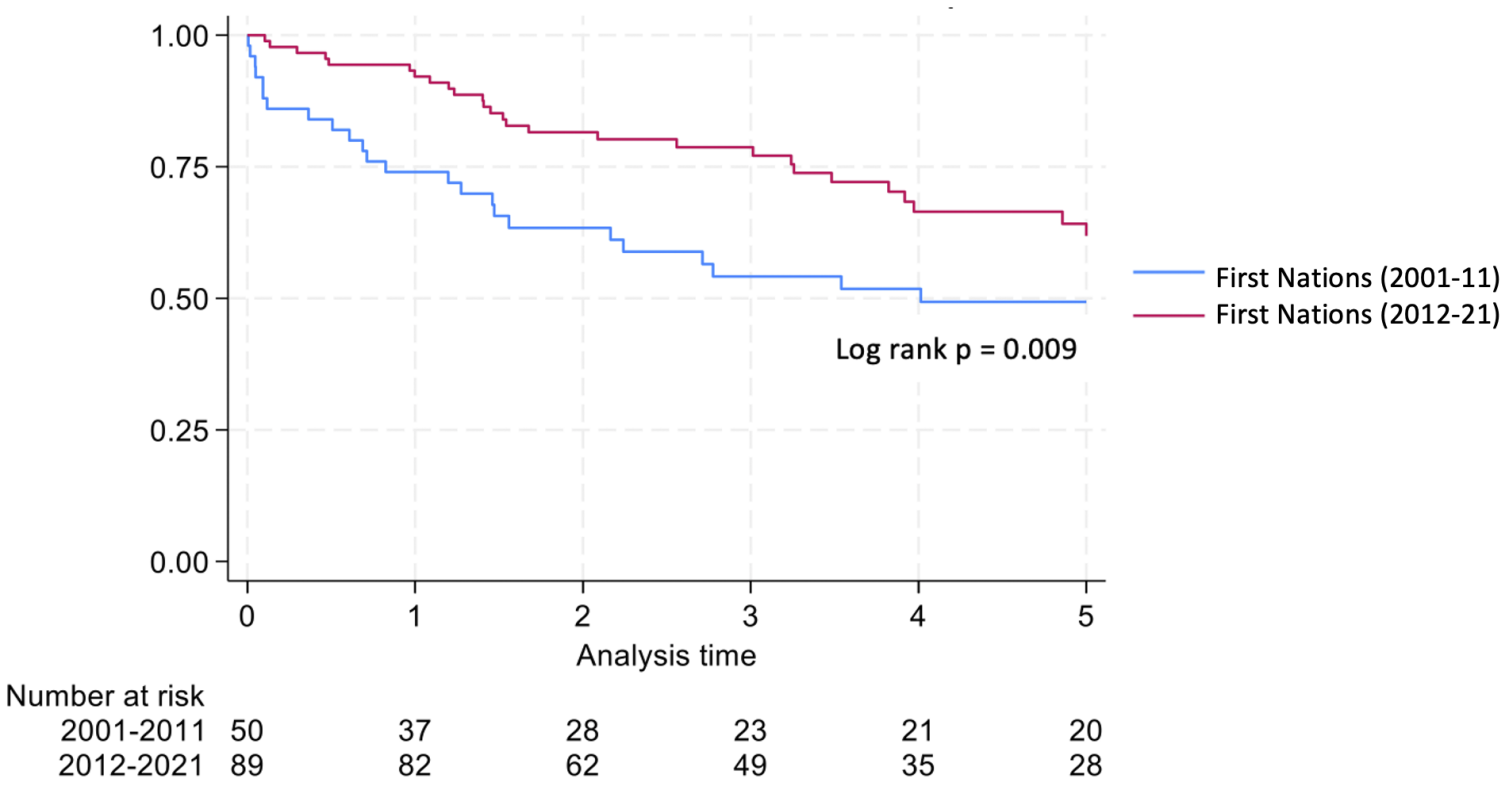
Graft survival for first nations transplant recipients.

**Figure 2:**
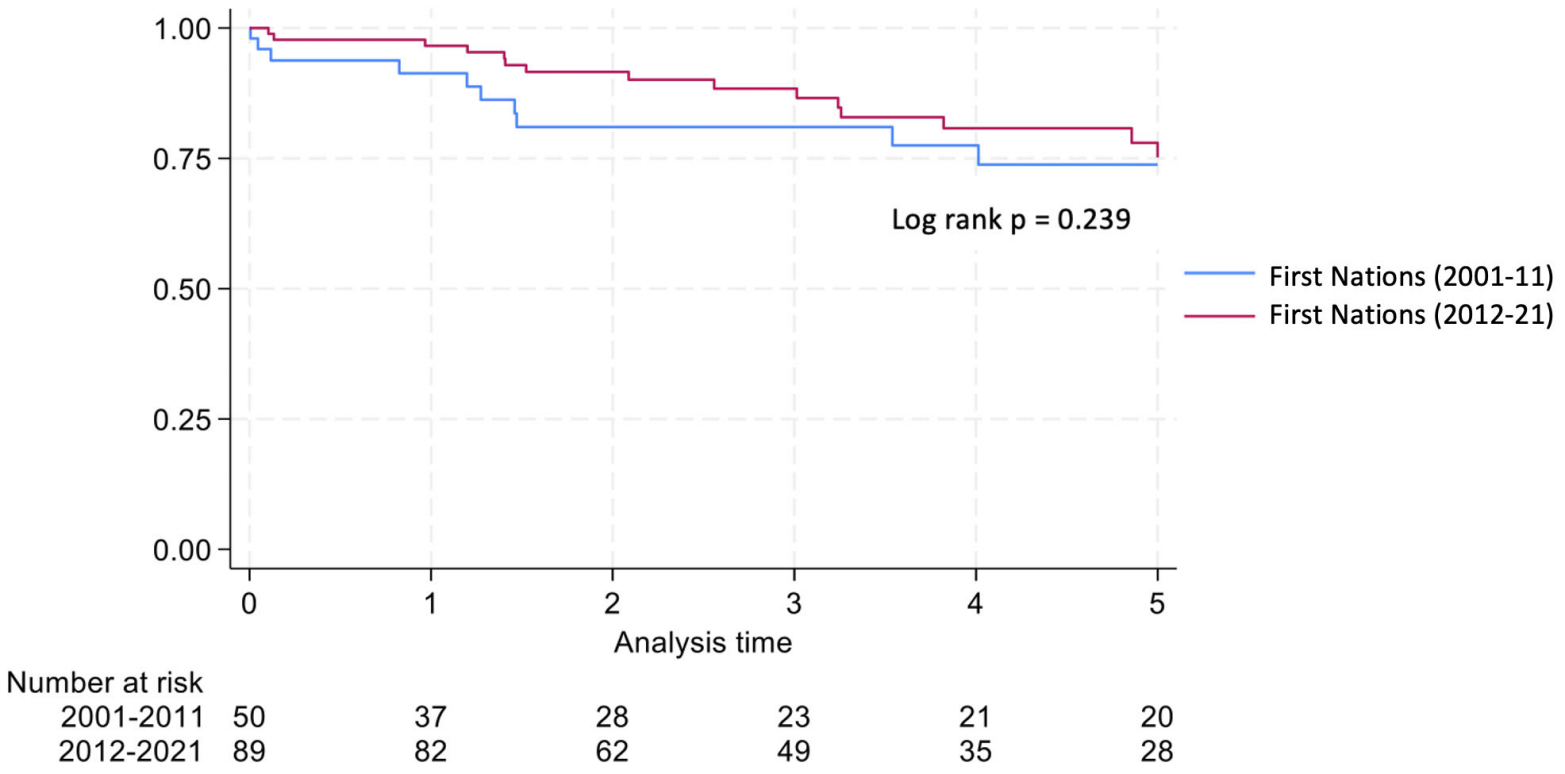
Death censored graft survival for first nations transplant recipients.

**Figure 3:**
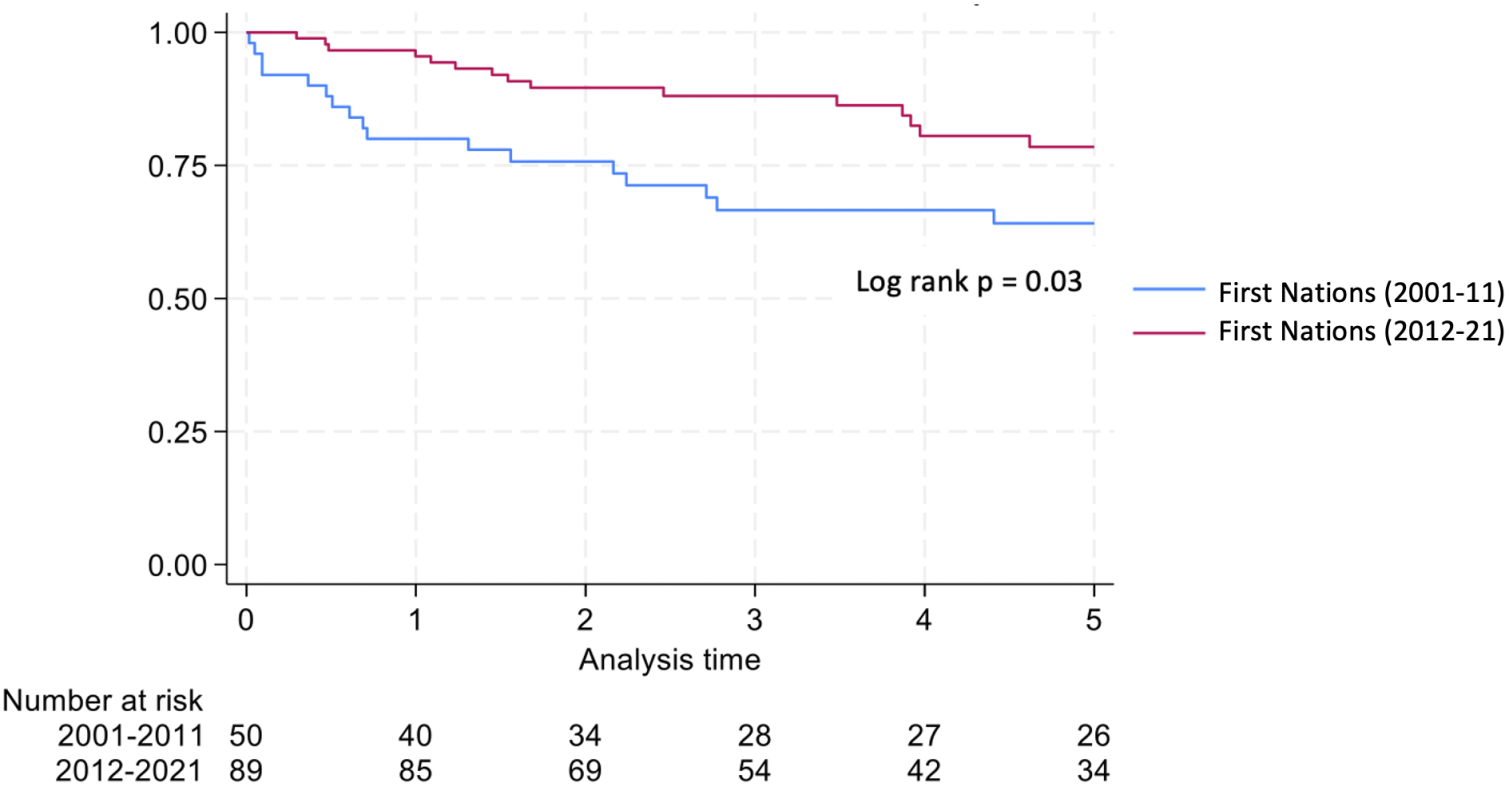
Patient survival for first nations transplant recipients.

Graft survival among First Nations Australians in the 2001–2011 era was 76%, 68%, and 46% at 1, 2 and 5 years, respectively (**Table 2**, **Figure 1**). Death censored graft survival was 92%, 84% and 80% at the same time intervals, while patient survival was 80%, 76%, and 66% (**Table 2**, **Figure 2**). Between 2012-2021, graft survival was significantly better, with rates of 92.1%, 82%, and 69.7% at 1, 2 and 5 years, respectively (p=0.009) (**Table 2**, **Figure 3**). Patient survival also improved (95.5%, 89.9% and 83.1% at 1,2 and 5 years, p=0.30), but death censored graft survival rates were similar (**Table 2**, **Figures 2**, **3**).

**Table 2:** Patient and graft survival outcomes.

Results	First nations patients			Non-indigenous	
	2001-2011 (n=50)	2012-2021 (n=89)	p*	2012-2021 (n=42)	p**
Death censored graft survival (0–5 years)					
1 year	92%	96%	LR p=.239	100.0%	LR p=.172
2 year	84%	92.10%		97.6%	
5 year	80%	84.20%		90.5%	
Years at risk	140.1	285.9		170.0	
Grafts lost	10	14		4	
Graft loss rate (per 100 patient-years)	7.1	4.9		2.4	
Graft survival (0–5 years)					
1 year	76.0%	92.1%	LR p=.009	100.0%	LR p=.002
2 year	68.0%	82.0%		97.6%	
5 year	46%	69.7%		90.5%	
Years at risk	140.1	285.9		170.0	
Grafts lost	24	26		4	
Graft loss rate (per 100 patient-years)	17.1	9.1		2.4	
Patient survival (0–5 years)					
1 year	80%	95.5%	LR p=.030	100.0%	LR p=.004
2 year	76%	89.9%		100.0%	
5 year	66%	83.1%		97.6%	
Years at risk	165.0	311.5		181.3	
Number of deaths	17	15		1	
Mortality rate (per 100 patient-years)	10.3	4.8		0.6	
Rejection (0–2 years)					
No of patients with rejection (0–2 years)	25 (50%)	29 (32.6%)	.043	9 (21.4%)	.190
TCMR (0–2 years)	98%	51.7%	<.001	66.7%	.711
ABMR (0–2 years)	0.0%	13.8%	.128	11.1%	.555
Mixed rejection (both TCMR and ABMR, 0–2 years)	2.0%	34.5%	0.052	22.2%	.230
Hospitalizations (0–2 years)					
Mean hospitalizations	5.1	8.7	<.001	4.8	<.001
Mean length of stay (days)	12.6	5.5	.001	4.1	.004
Mean infectious hospitalizations	38.8%	33.6%	.049	33.0%	.105
Patients hospitalized for cytomegalovirus disease	7 (14%)	8 (9.0%)	.362	4 (9.5%)	.920
Cause of graft loss					
Death	16 (32%)	13 (14.6%)	.016	0	.009
Acute rejection	1 (2%)	5 (5.6%)	.313	3 (7.1%)	.727
Chronic rejection	5 (10%)	3 (3.4%)	.107	0	.230
Infection	3 (6%)	4 (4.5%)	.697	0	.162
Non-adherence to medication	4 (8%)	6 (6.7%)	.779	0	.085
Transplant ischemia	2 (4%)	0	.057	1 (2.4%)	.144
Cause of death					
Infection	15 (30%)	7 (7.9%)	<.001	0	.061
Cardiovascular	3 (6%)	3 (3.4%)	.465	0	.230
Other	3 (6%)	6 (6.7%)	.865	1 (2.4%)	.298

LR, Log rank; ABMR, Antibody Mediated Rejection; TCMR, T-Cell Mediated Rejection.

*Comparison between First Nations transplants in the 2 eras.

**Comparison between First Nations and non-indigenous patients in 2012–2021 era.

There was a significant reduction in death with a functioning graft in 2012–21 compared with 2001-2011 (32% vs. 14.6% p=0.016) (**Table 2**). Otherwise, causes of graft loss were similar over time. The proportion of deaths due to infection were significantly lower in 2012–2021 compared to 2001-2012.

**Figure 4:**
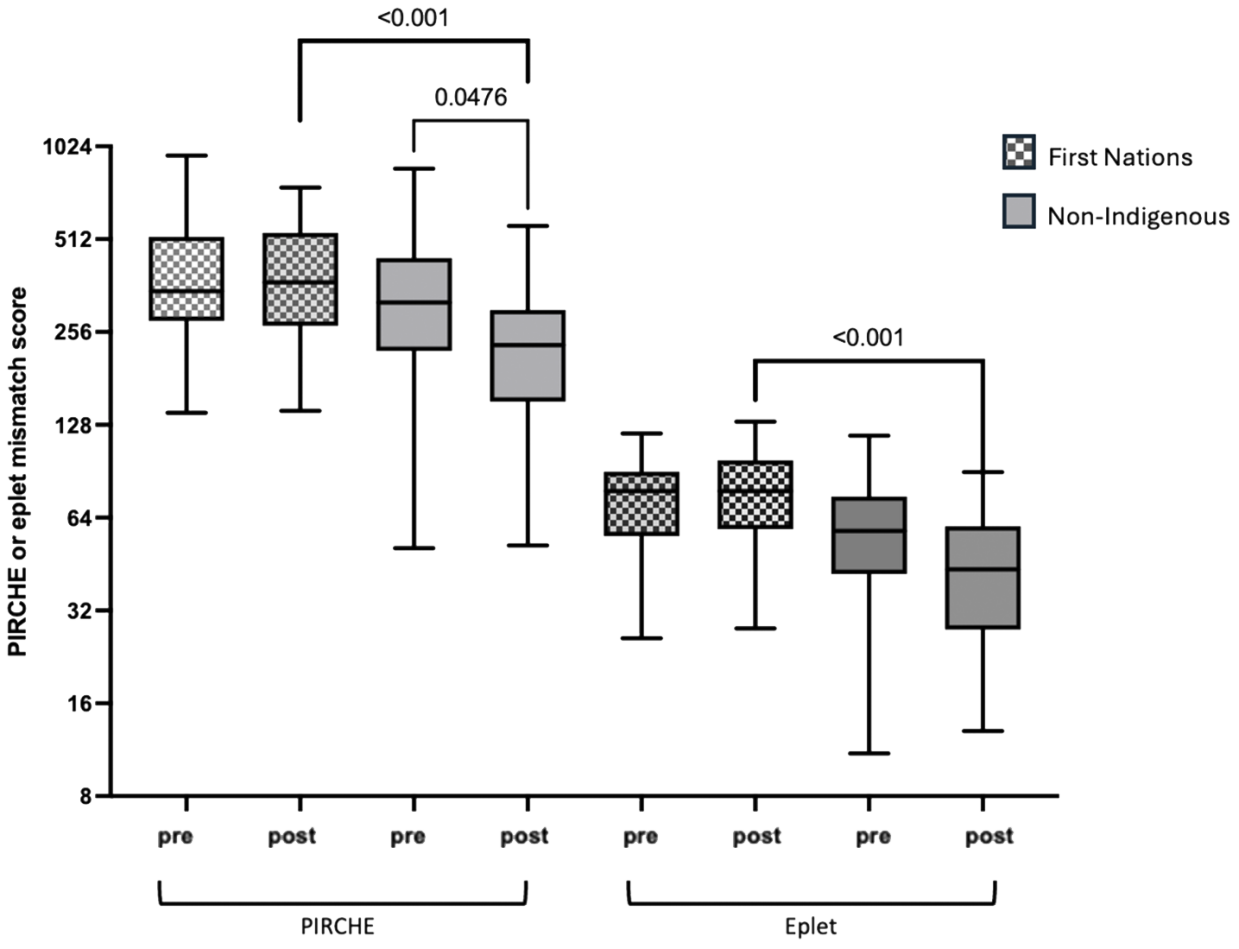
Change in PIRCHE and eplet scores for first nations and non-indigenous recipients pre and post the introduction of cPRA-based allocation.

When comparing First Nations to non-Indigenous recipients, the incidence of diabetic nephropathy as the primary kidney disease and as a comorbidity was significantly higher in the First Nations group (56.2% vs. 11.9%, p<0.001 and 69.7% vs. 19%, p=<0.001). First Nations recipients were also more likely to have completely HLA mismatched transplants (69.7% vs. 15.1%, p<0.001; **Table 1**) and higher eplet mismatch loads and PIRCHE scores (77 vs. 53 and 407 vs. 289 respectively, p<0.001; **Table 1**). Post-implementation of the altered process of assigning cPRA, the median HLA mismatches remained at 6 in First Nations recipients but improved from a median of 5 to 3 in non-Indigenous recipients (p=0.009). While there was no improvement in eplet or PIRCHE scores in First Nations recipients, PIRCHE score improved in non-Indigenous recipients (p=0.0476; **Figure 4**). The gap in eplet and PIRCHE scores between First Nations and non-Indigenous recipients widened following the change to use of cPRA (p<0.001; **Figure 4**).

**Figure 5:**
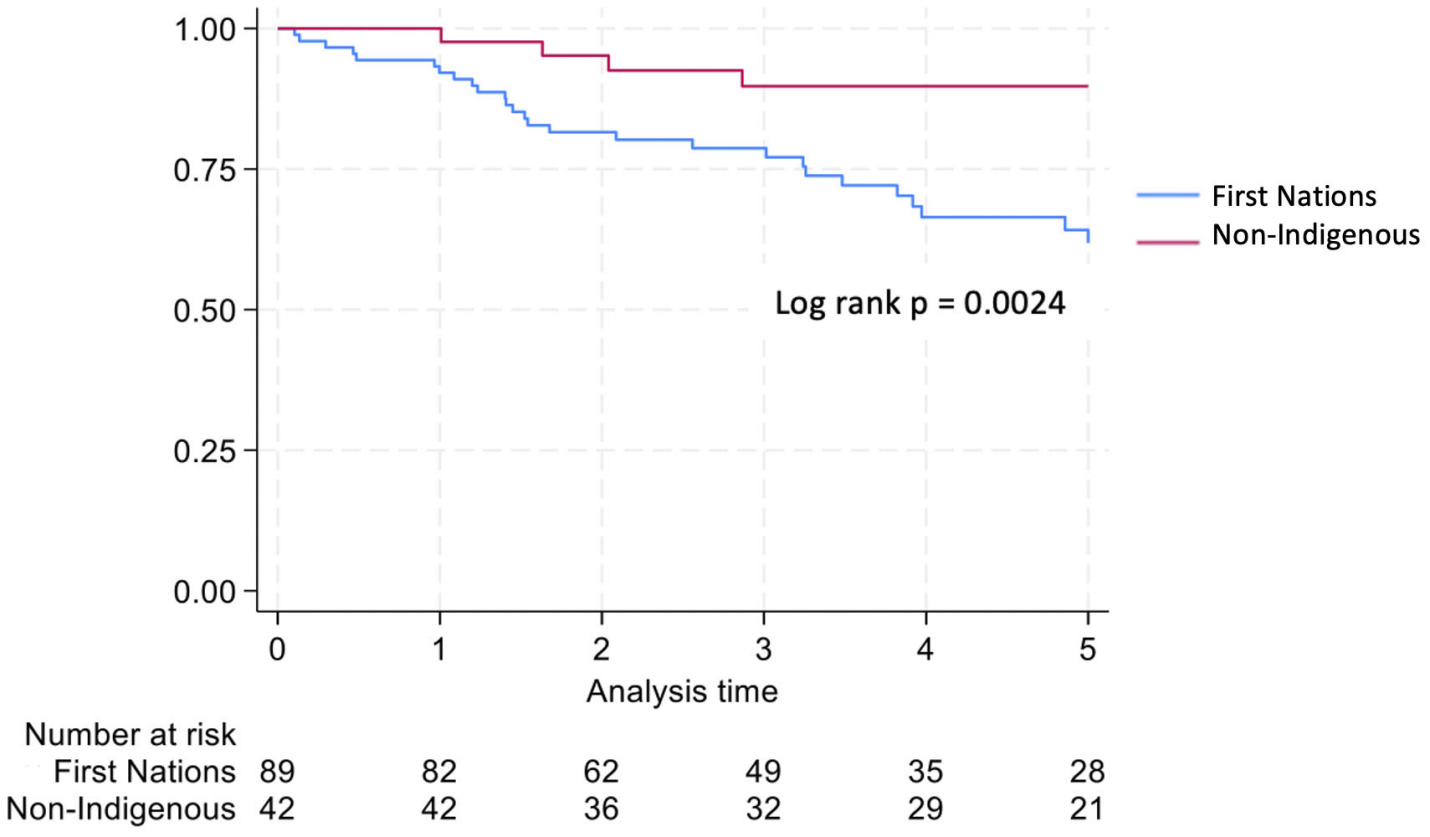
Graft survival first nations vs. non-indigenous transplant recipients.

**Figure 6:**
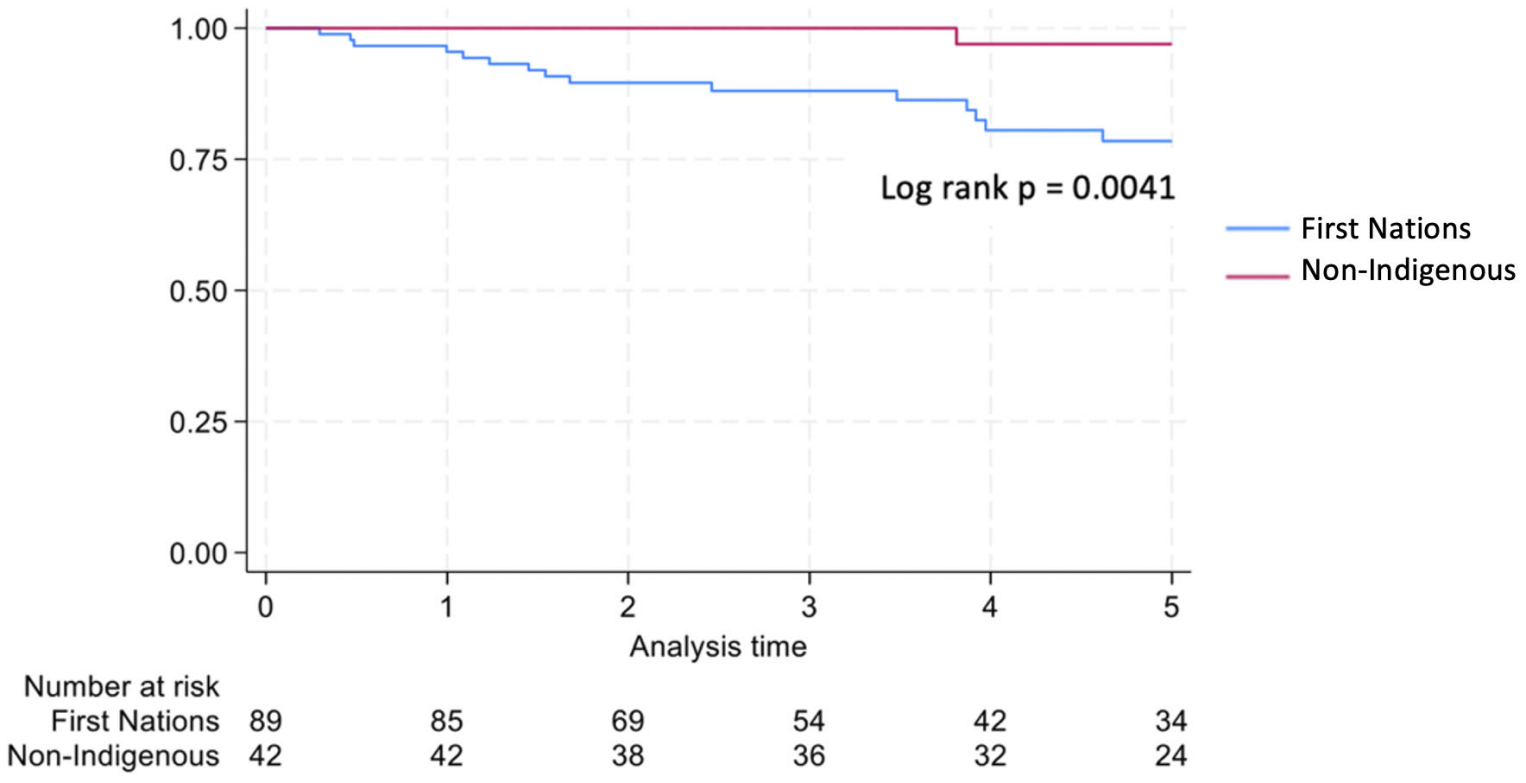
Patient survival first nations vs. non-indigenous transplant recipients.

**Figure 7:**
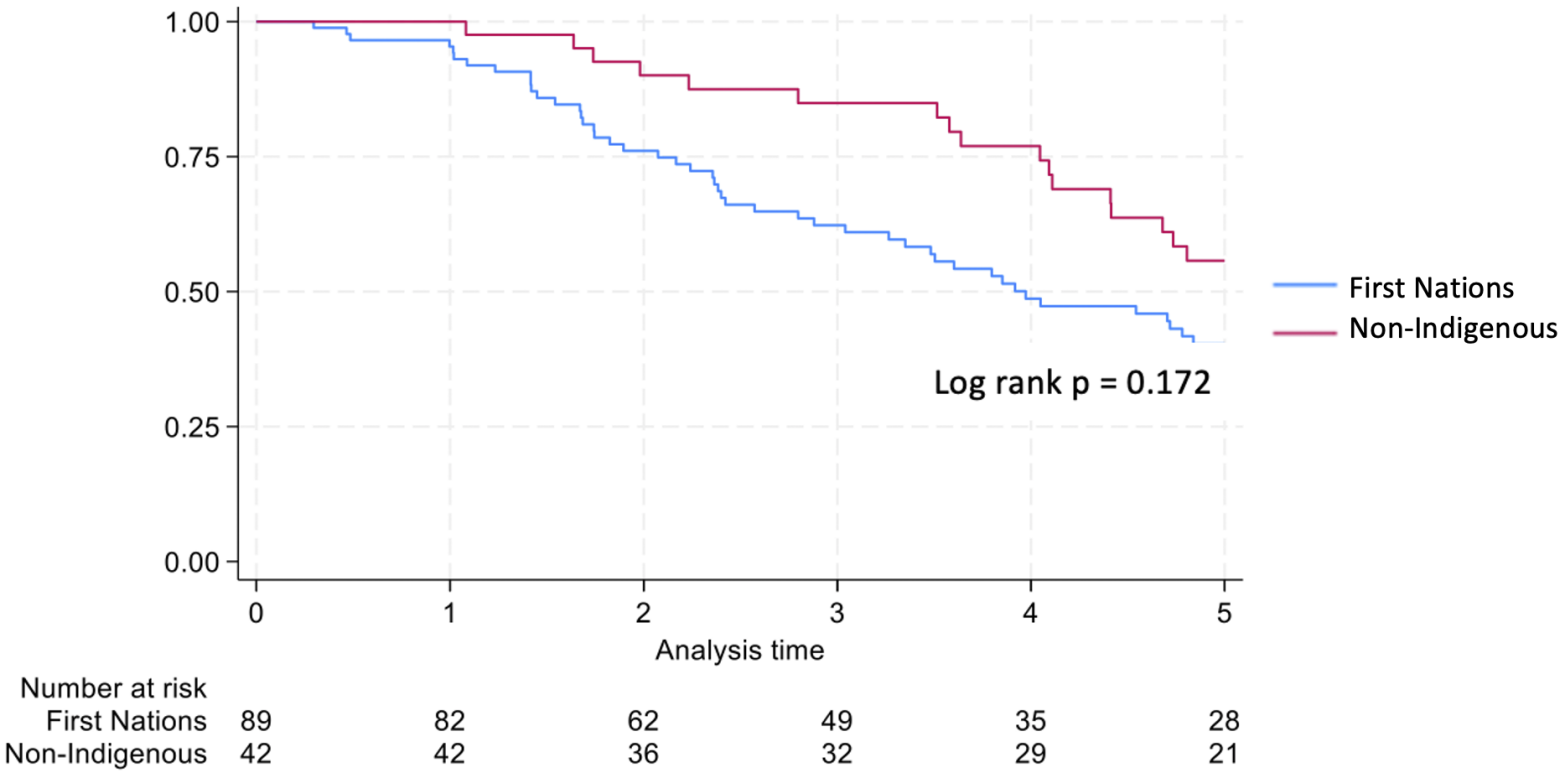
Death censored graft survival first nations vs. non-indigenous transplant recipients.

Non-Indigenous recipients had significantly less hospitalizations (4.8 vs. 8.7, p<0.001) and length of stay days (4.1 vs. 5.1, p=0.004) compared to their First Nations counterparts. The proportion of admissions for infective complications was not statistically different between the groups. Rates of rejection were also similar. Both graft survival (p=0.002) and patient survival (p=0.004) were significantly higher in non-Indigenous compared with First Nations recipients (**Table 2**, **Figures 5**, **6**). In contrast, death-censored graft survival was not significantly different between the two groups (**Table 2**, **Figure 7**). Multivariate analysis did not reveal significant differences.

**Figure 8:**
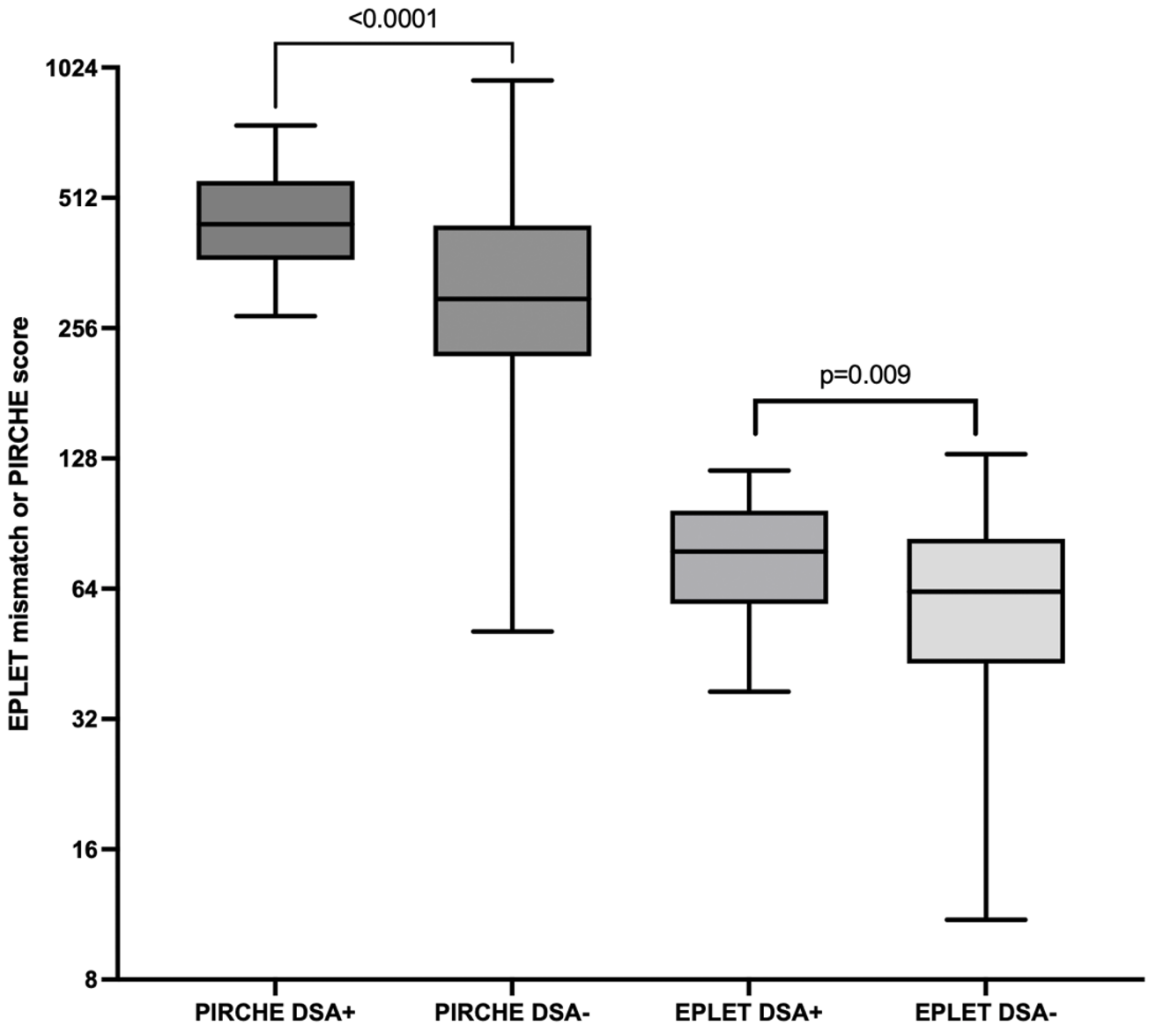
Combined dataset of first nations and non-indigenous into either DSA positive or negative. For PIRCHE, the area under the ROC curve for dnDSA formation was 0.75 (95% CI 0.65-0.84, p < 0.001), with a threshold of <275 yielding 96% specificity and 42% sensitivity. For eplet mismatch, the area under the ROC curve was 0.67 (95% CI 0.57-.078, p = 0.009) with threshold <54 yielding 96% specificity and 41% sensitivity.

**Figure 9:**
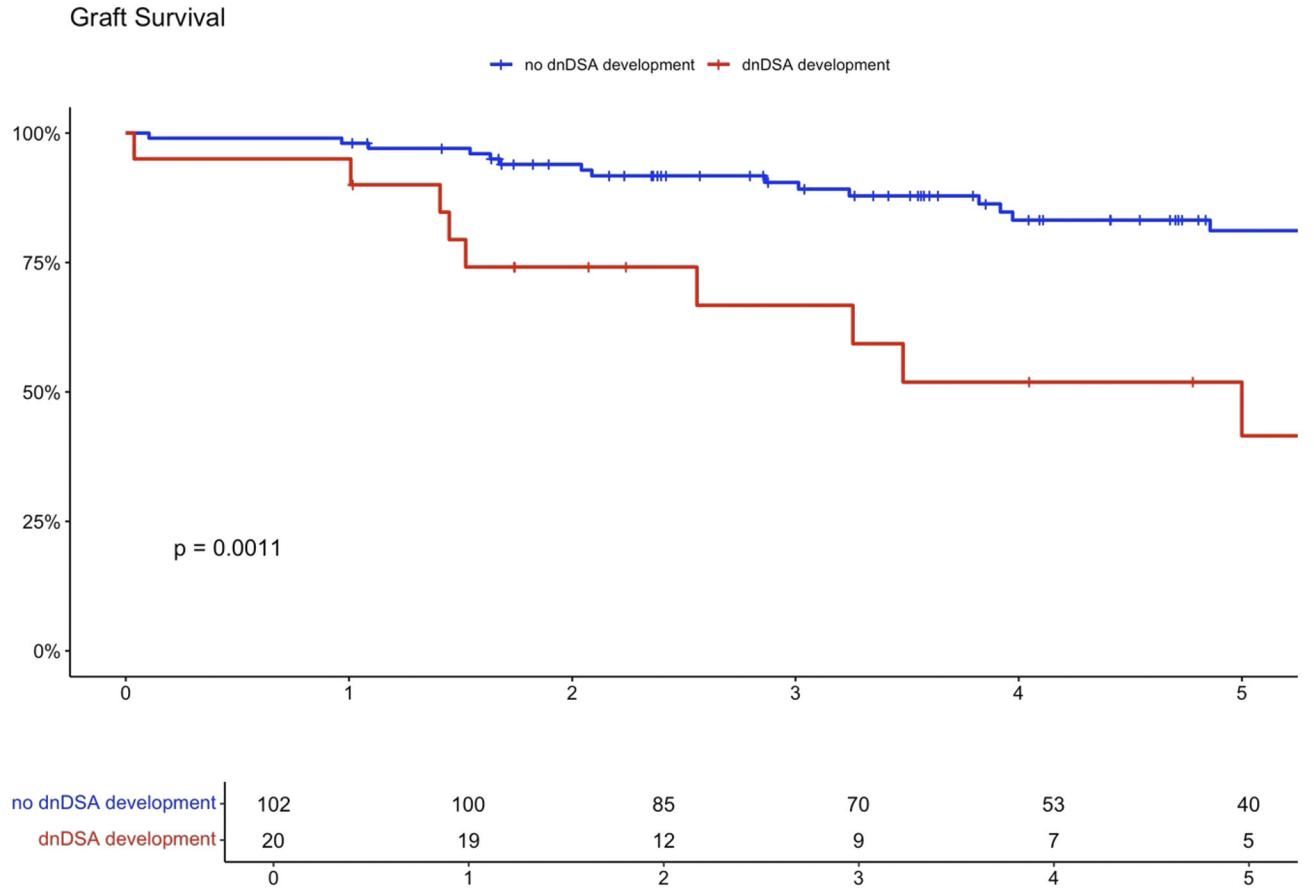
Combined cohort graft survival with dnDSA positive vs. negative.

**Figure 10:**
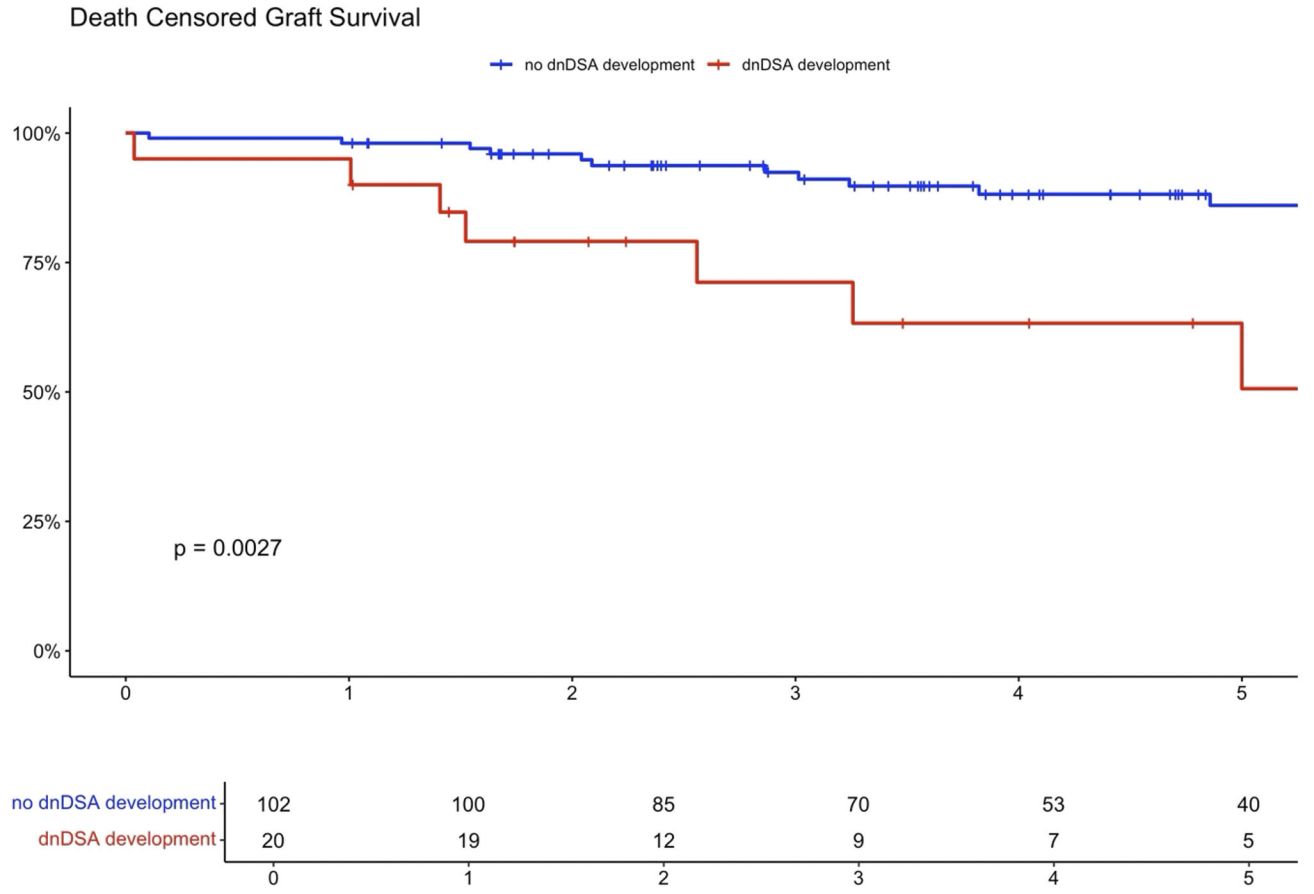
Combined cohort death censored graft survival dnDSA positive vs. negative.

Higher eplet mismatch loads and PIRCHE scores were not associated with graft survival, patient survival, or time to rejection among First Nations Australians (**Supplementary Figures S1**–**S5**). However, in the combined 2012–21 cohort, ROC curve analysis identified a PIRCHE score <275 and an eplet mismatch <54 as thresholds associated with a lower incidence of dnDSA development (**Figure 8**). Across the entire 2012–2021 period, 15% of First Nations recipients received a <54 eplet mismatch kidney (median HLA mismatch 4) and 28% received a kidney with a PIRCHE score of <275 (median HLA mismatch 6). In contrast, following revision of the cPRA-based allocation in early 2021, no First Nations recipients received a graft with PIRCHE score less than 237 or eplet mismatches less than 55. While graft and death censored graft survival were lower in First Nations recipients who developed dnDSA, these differences did not reach statistical significance (p=0.11 and 0.13 respectively; **Supplementary Figures S6**, **S7**). However, across the combined 2012–2021 cohort, dsDNA development was significantly associated with poorer graft and death censored graft survival (**Figures 9**, **10**).

## Discussion

This study demonstrates significant improvements in graft and patient survival outcomes for First Nations kidney transplant recipients from the Northern Territory of Australia since 2012. These improvements have occurred despite worsening HLA matching and increasing rates of diabetic nephropathy.

Though the number of hospitalizations for First Nations Australians almost doubled in the 2012–2021 time frame, the length of stay more than halved. This likely reflects closer follow up of patients and earlier hospitalizations with lower severity of disease.

The reduction in the rates of organ rejection within the first 2 years, particularly T cell mediated rejection, may be secondary to altered immunosuppressive protocols, particularly greater use of ATG in the later era. In addition, an increase and greater stability in the local nephrology workforce are likely to be contributing factors. Graft and patient survival also improved between eras; however, death-censored graft survival showed no significant change, indicating that the observed improvement in graft outcomes was mainly due to enhanced patient survival, rather than an independent improvement in graft survival. While infection remained the leading cause of deaths in both eras, the proportion of infective deaths decreased markedly, from 30% between 2001–2011 to 7.9% between 2012-2021. This reduction may be linked to the lower rates of rejection, which in turn would have decreased the need for additional immunosuppressive treatment.

Consistent with previous studies, we observed poorer graft and patient outcomes in First Nations compared with non-Indigenous Australian kidney transplant recipients. Rogers et al. reviewed all kidney transplant outcomes in the NT from July 1984 till June 2004 and showed significantly worse graft survival in the First Nations group (8). The majority of grafts were lost due to patient death, with infection identified as the leading cause of mortality. In an examination of nearly 8000 Australians receiving primary grafts over the period 2000 to 2012, 3% of whom were First Nations Australians, 5-year graft and patient survival rates were approximately 25% lower in First Nations group (4). Our study similarly demonstrated significantly lower 5-year patient survival for First Nations kidney transplant recipients. However, encouragingly, the survival gap between First Nations and non-Indigenous groups appears to have narrowed over time, to 14.5% in our cohort. While graft survival was also lower in the First Nations group, death-censored graft survival rates were similar, suggesting that the differences in graft outcomes were largely driven by higher mortality rates among First Nations recipients. 5-year patient and graft survival outcomes in First Nations recipient in the 2012–21 cohort was comparable to outcomes for First Nations recipients nationally during a similar time frame (2013-22) (9). As in Rogers et al’s study, most graft losses in First Nations recipients were due to patient death, with infection being the primary cause of mortality.

This underscores the importance of not only continuing efforts to optimize immunosuppression exposure for First Nations recipients, but also addressing other factors that contribute to increased infection risk, such as poor diabetes control, inadequate nutrition, substandard housing, and insufficient sanitation infrastructure (14). Moreover, targeted strategies to optimize the management of co-morbidities, particularly cardiovascular disease and its associated risk factors, are crucial for narrowing the persistent gap in graft and patient outcomes. Whilst outcomes in the acute post-transplant period are improving, sustaining this long term, requires addressing social determinants, access to healthcare and tailoring care in a culturally safe manner (9, 15–18). The National Indigenous Kidney Transplantation Taskforce was established to improve access to and outcomes post transplantation for First Nations Australians in 2019. Initial recommendations made by the taskforce included immediate improvements to access and services, ongoing secretariat to monitoring and progress of transplantation equity and, investigation into additional measures to address drivers of inequity (19). Our research further strengthens the importance of following these recommendations to bridge the gap between post transplantation outcomes between First Nations and non-indigenous Australians.

First Nations transplant recipients received grafts with less favorable HLA and molecular matching compared to non-Indigenous recipients, with this disparity increasing after changes were made to the process of assigning cPRA and the introduction of a new allocation algorithm. Despite this, eplet mismatches and PIRCHE scores did not have a significant impact on First Nations graft outcomes. This may be reflective of the study being underpowered for this outcome, or because that the overall degree of HLA and molecular mismatches were high with low variability in First Nations recipients, making it difficult to detect the effect of small differences in mismatches on graft survival. In the combined cohort, a high level of molecular mismatches was associated with dnDSA formation, which, in turn, was associated with poor graft outcomes.

A major limitation of the study was its retrospective nature, with substantial data collected from medical records. Other limitations include the small sample size and the lack of biopsy data to confirm primary kidney disease for some patients. For example, not all cases of diabetic nephropathy were biopsy proven, with the impression of the treating nephrologist was used in these instances.

## Conclusions

Graft and patient survival among First Nations Australians in the NT have improved over the last decade. This is despite rising rates of diabetic nephropathy and increasing HLA mismatches over time. Compared to non-Indigenous Australians, however, there remains a marked disparity in post-transplant outcomes. First Nations Australians were likely to have less favorable molecular matching, and although this was not directly associated with time to rejection or graft survival, *de novo* DSA formation was associated with worse graft outcomes in our analysis of the combined cohort. This suggests that there may be scope to offer First Nations people lower PIRCHE and eplet score kidneys as a means of improving their transplant outcomes.

## Data Availability

The raw data supporting the conclusions of this article will be made available by the authors, without undue reservation.
