# Supplementary material for: Outcomes post kidney transplantation amongst First Nations Australians in the Northern Territory
Source: Front Nephrol. 2025 Oct 9;5:1677030. doi: 10.3389/fneph.2025.1677030 (PMC12545150; doi:10.3389/fneph.2025.1677030)
Supplement: Supplementary file 1 [file DataSheet1.pdf]

Supplementary figures

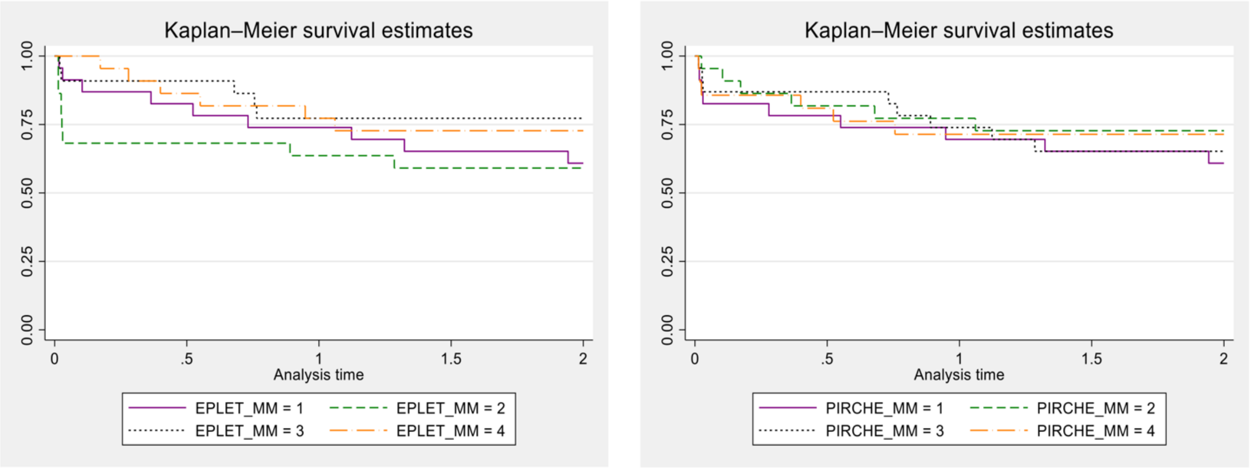

Figure S1: Time to Rejection by Eplet MM and PIRCHE score quartiles for First Nations transplant recipients 2012-21 (lowest quartile labelled 1, highest, 4)

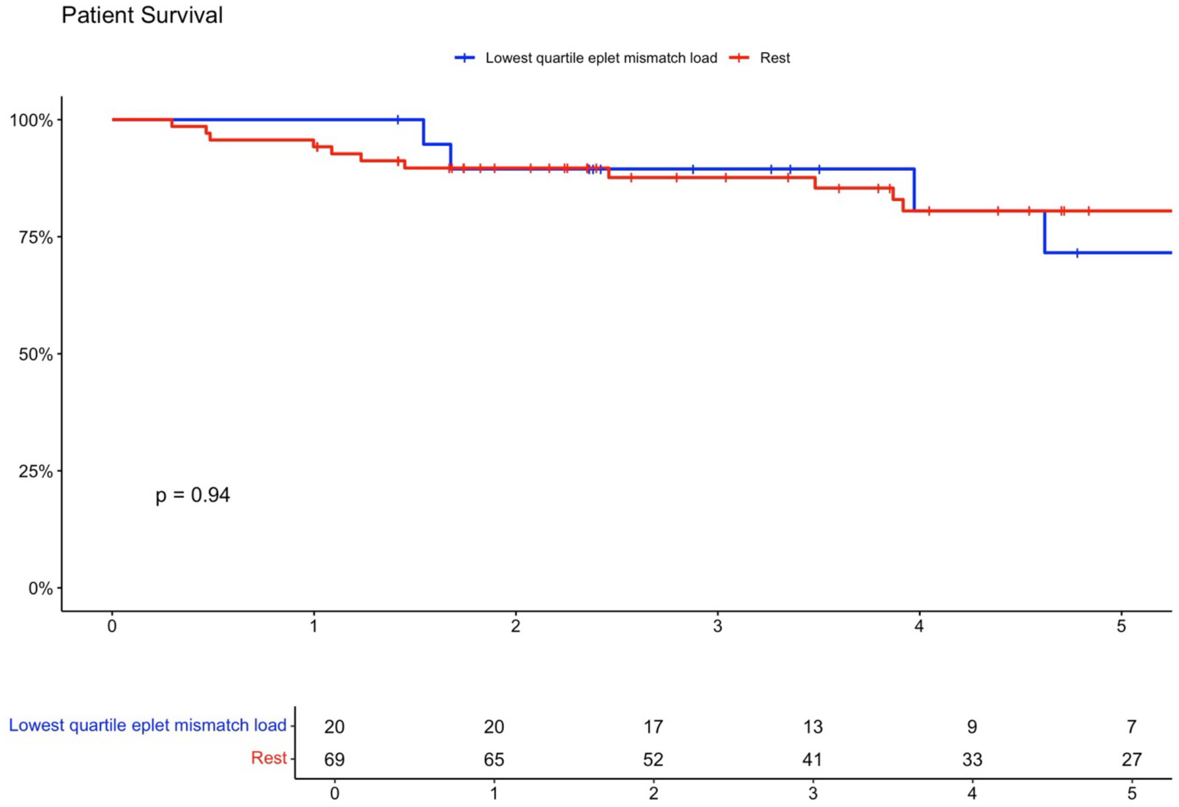

Figure S2: Patient survival for First Nations transplant recipients 2012-21, lowest quartile eplet mismatch loads vs. rest

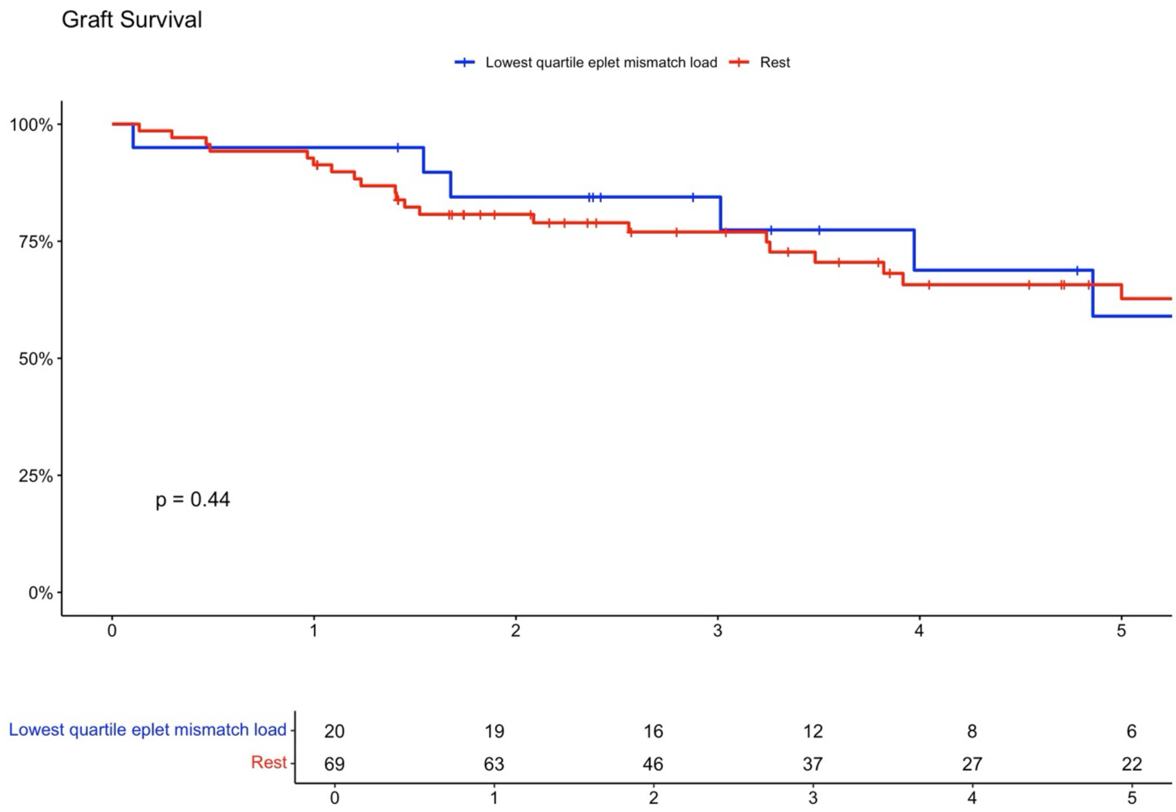

Figure S3: Graft survival for First Nations transplant recipients 2012-21, lowest quartile eplet mismatch loads vs. rest

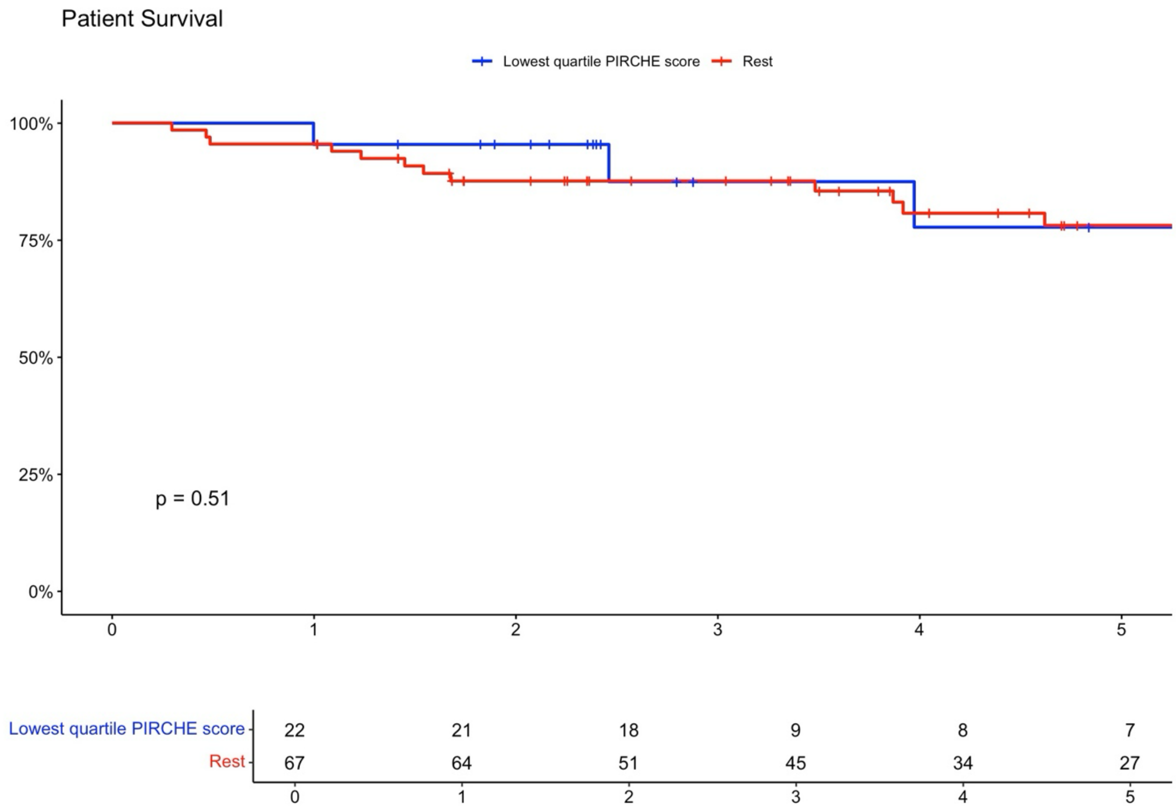

Figure S4: Patient survival for First Nations transplant recipients 2012-21, lowest quartile PIRCHE score vs. rest

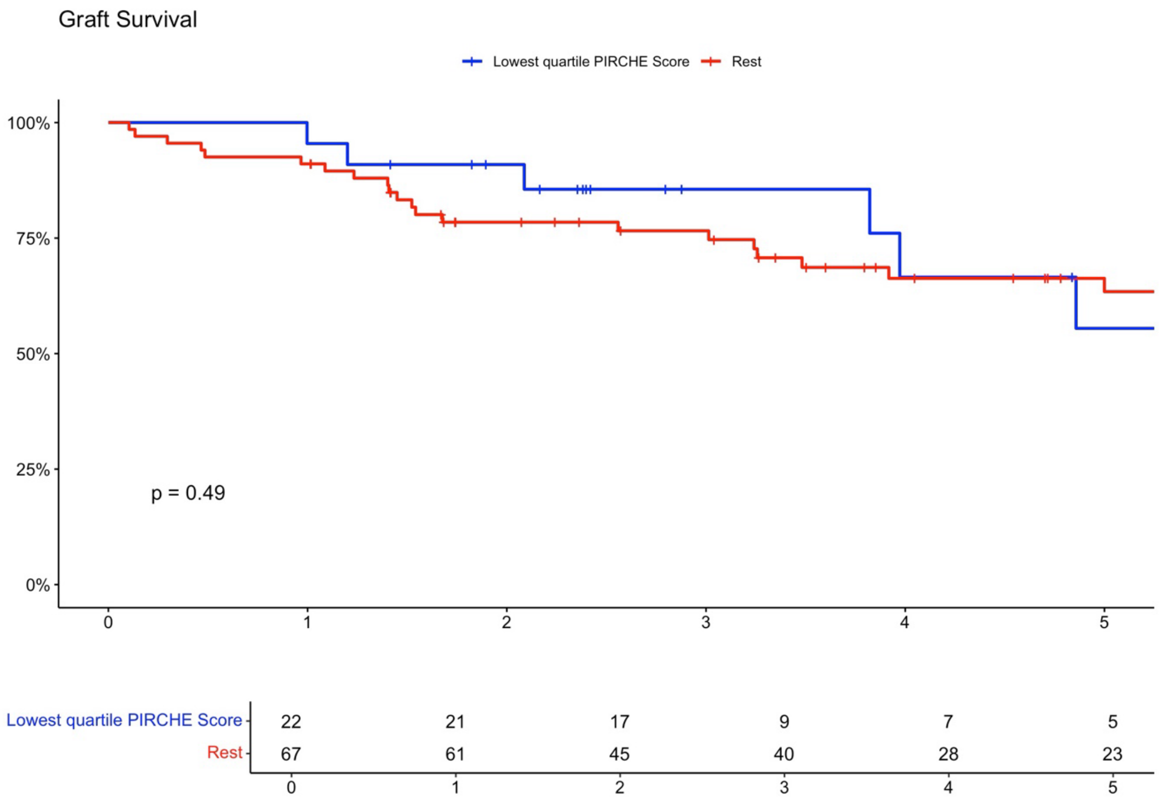

Figure S5: Graft survival for First Nations transplant recipients 2012-21, lowest quartile PIRCHE score vs. rest

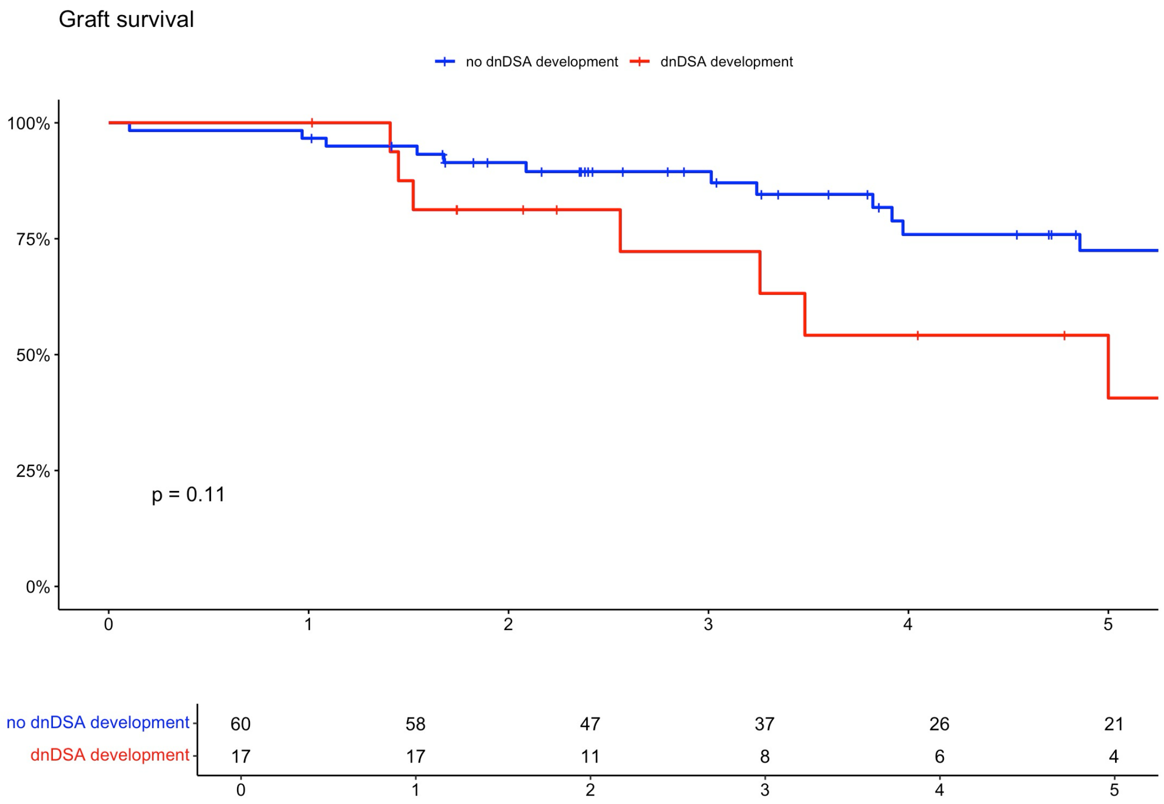

Figure S6: First Nations graft survival with dnDSA positive vs. negative

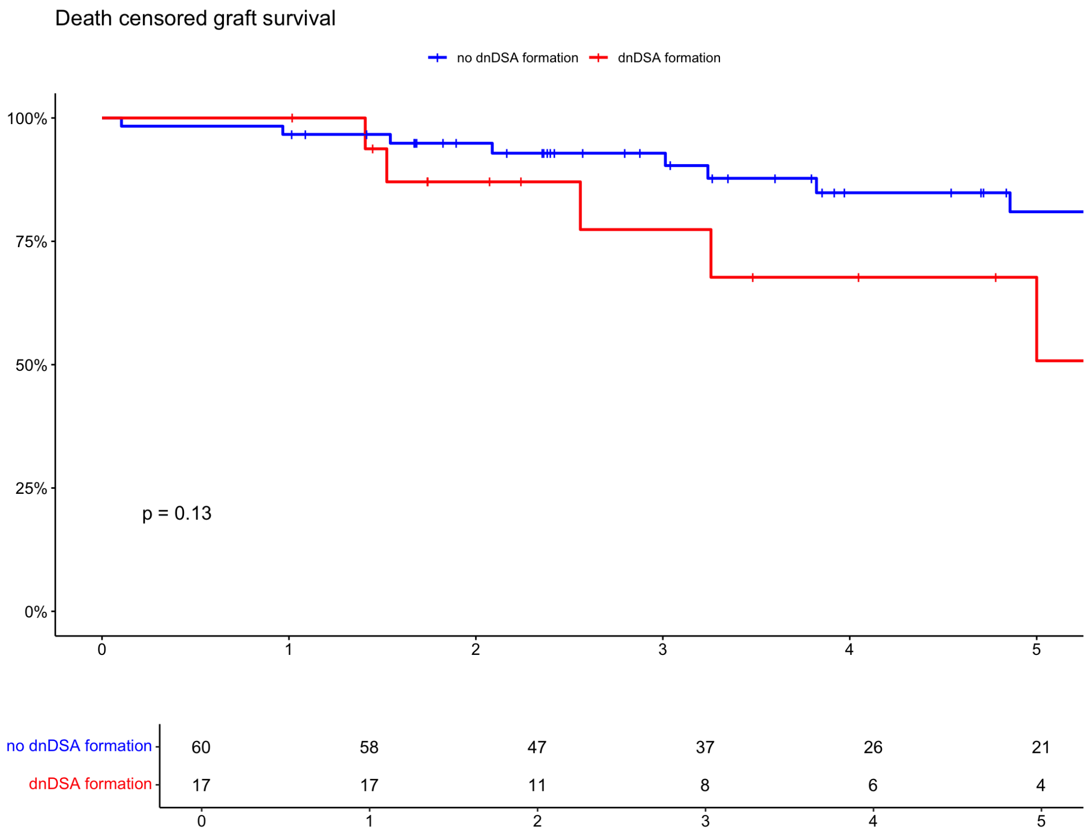

Figure S7: First Nations death censored graft survival with dnDSA positive vs. negative
